# Inhibition of the integrated stress response reverses oxidative stress damage-induced postoperative cognitive dysfunction

**DOI:** 10.3389/fncel.2022.992869

**Published:** 2022-09-21

**Authors:** Linhao Jiang, Rui Dong, Minhui Xu, Yujia Liu, Jiyan Xu, Zhengliang Ma, Tianjiao Xia, Xiaoping Gu

**Affiliations:** ^1^Department of Anesthesiology, Affiliated Drum Tower Hospital of Medical School of Nanjing University, Nanjing, China; ^2^Medical School, Nanjing University, Nanjing, China; ^3^Jiangsu Key Laboratory of Molecular Medicine, Nanjing University, Nanjing, China; ^4^State Key Laboratory of Pharmaceutical Biotechnology, Nanjing University, Nanjing, China

**Keywords:** oxidative stress damage, postoperative cognitive dysfunction, ISRIB, the integrated stress response, eIF2α

## Abstract

Postoperative cognitive dysfunction (POCD) is a common complication following anesthesia and surgery that might lead to a decline in learning and memory. Oxidative stress damage is one of the pathogenic mechanisms underlying POCD. Recent studies had shown that the integrated stress response (ISR) is closely related to oxidative stress. The core response of the ISR is phosphorylation of eIF2α. Various cellular stress stimuli trigger activation of eIF2α kinases, thus causing phosphorylation of eIF2α. ISR is associated with many neurodegenerative diseases; however, the relationship between POCD and ISR has not been defined. In the present study, the tibias in 4-month-old male C57BL/6 mice were fractured under isoflurane anesthesia to establish the POCD animal model. Cognitive function was assessed by fear conditioning tests and the Y-maze from 3 to 14 days post-surgery. Western blot was used to determine the levels of PeIF2α, eIF2α, ATF4, GADD34, CHOP, BDNF, proBDNF, and p-NR2B expression. The levels of reactive oxygen species (ROS), superoxide dismutase (SOD), and malondialdehyde (MDA) were measured to determine oxidative stress in hippocampal tissues. After tibial fracture surgery in mice, the hippocampus had increased levels of PeIF2α, ATF4, GADD34, and CHOP protein, ROS-positive cells, and average fluorescence intensity, SOD activity was decreased, and the MDA level was increased. The ISR inhibitor, ISRIB, reduced the levels of PeIF2α, ATF4, GADD34, and CHOP protein, and alleviated oxidative stress in the hippocampus of POCD mice. Moreover, ISRIB ameliorated cognitive dysfunction in POCD mice. Our findings suggested that targeting ISR may represent an effective approach to combat POCD.

## Introduction

Postoperative cognitive decline (POCD) is a clinical phenomenon characterized by cognitive impairment in patients following anesthesia and surgery, accompanied by a series of negative outcomes, such as changes in mood and personality (Lin et al., 2020). The mechanisms underlying POCD include oxidative stress damage (Netto et al., 2018), neuroinflammation (Luo et al., 2019), and blood-brain barrier damage (Zhang et al., 2016). Our previous studies showed that oxidative stress plays an important role in the development of POCD (Song et al., 2019; Gong et al., 2020; Liu et al., 2020). Numerous cellular mechanisms associated with oxidative stress damage are involved in the occurrence of various types of neurodegenerative diseases. Oxidative stress damage is accompanied by mitochondrial dysfunction (Lin and Beal, 2006). It has recently been shown that integrated stress response (ISR) regulated oxidative stress in mitochondria (Koncha et al., 2021; Zhang et al., 2021).

The ISR is an evolutionarily-conserved intracellular signaling network. The ISR can help cells restore their homeostasis under various stress stimuli, such as oxidative stress, inflammation, and endoplasmic reticulum stress (Pakos-Zebrucka et al., 2016). These stress stimuli activate kinases, including PERK, GCN2, PKR, and HRI, which cause phosphorylation of initiation factor 2α (eIF2α; Costa-Mattioli and Walter, 2020). The core of ISR is the phosphorylation of eIF2α (Bond et al., 2020). The ISR causes reprogramming of global protein synthesis and triggers specific mRNA translation, such as activating transcription factor 4 (ATF4). ATF4 then initiates a series of signaling cascades. Recent studies have suggested that ISR is involved in the pathogenesis of many neurodegenerative diseases (Chang et al., 2022), such as Alzheimer’s disease (Oliveira and Lourenco, 2016; Hu et al., 2022), amyotrophic lateral sclerosis (ALS; Ghadge et al., 2020), and Down syndrome (Zhu et al., 2019). It has been suggested that ISR had an important mechanistic connection with neurocognition (Pakos-Zebrucka et al., 2016), but the relationship between ISR and POCD is unclear.

Therefore, in this study, we determined whether ISR occurred in POCD mice and showed the effect of ISR on the oxidative stress level and cognitive function in a POCD murine model by targeting the ISR. Our findings enriched the pathogenic mechanism underlying POCD.

## Materials and Methods

### Animals

Four-month-old male C57BL/6 mice (Weitong Lihua Experimental Animal Technology Co., Ltd., China) were housed under standard conditions in a 12-h light/12-h dark cycle (lights on at 8 a.m.) at a constant temperature (22 + 1°C) and relative humidity (50% ± 10%). Animals were housed in groups and allowed free access to standard mouse chow and water *ad libitum*. The experiments commenced after all of the animals had acclimated to the experimental environment for 2 weeks. All animal procedures were conducted following the Guidelines for Care and Use of Laboratory Animals of Nanjing University, and all efforts were made to minimize animal suffering. The study protocol was approved by the Ethics Committee (license no. 2020AE01110) of the Affiliated Drum Tower Hospital (Medical School of Nanjing University, Nanjing, China). A total of 280 mice were grouped using a random number table. The mice were divided into four groups, namely, the control plus vehicle group (C + Veh), the control plus ISRIB group (C + ISRIB), the surgery plus vehicle group (S + Veh), and the surgery plus ISRIB group (S + ISRIB).

### Tibial fracture surgery

We performed open tibial fracture surgery as previously described (Feng et al., 2017). Briefly, the tibia was fractured under isoflurane anesthesia (2.1% inspired concentration in 0.4 FiO_2_) and analgesia with buprenorphine (0.1 mg/kg s.c.). Under aseptic surgical conditions, the mice were shaved and the right tibia was disinfected. A median paw incision was then performed, followed by the insertion of a 0.38-mm pin in the tibial intramedullary canal. The soft tissues of the lower two-thirds of the tibia were lifted, and the periosteum was circumferentially stripped for a distance of 10 mm. Finally, an osteotomy was created under direct vision at the junction of the middle and distal one-third of the tibia with osteotomy scissors. After producing the fracture, the wound was irrigated and the skin sutured with 6–0 suture.

### Drug administration

ISRIB solution (SML0843; Sigma-Aldrich, USA) was made by dissolving 5 mg of ISRIB in 1 ml of dimethyl sulfoxide [(DMSO) D4540; Sigma] and 1 ml of PEG400 (P8530; Solarbio, Beijing, China). The solution was gently heated in a 40°C water bath and vortexed every 30 s until the solution was clear. ISRIB was injected intraperitoneally after tibial fracture surgery in mice, the dosage we gave was 7.5 mg/kg. The vehicle solution consisted of 1 ml DMSO and 1 ml PEG400.

### Behavioral assessments

Hippocampal-dependent place learning and memory were evaluated using the fear conditioning test (FCT) and Y-Maze test on post-surgery days 3, 7, and 14. Mice were placed in a chamber (25 × 25 × 25 cm; Panlab/Harvard Apparatus, Barcelona, Spain) for 3 min of habituation and free exploration of the context. Next, the mice were subjected to a conditioned stimulus (CS; 28 s, 4 kHz, 90 dB), followed by a foot-shock signal (2 s, 0.8 mA) from the floor in the chamber. The chambers were cleaned with 75% alcohol between each session. Both contextual and cue memory evaluation were performed the following day. For contextual tests, mice were placed in the same chamber for 3 min without foot shocks. For the cue tests, the chambers were modified to have an altered appearance, and the mice were exposed to the same CS cue without footshocks. The freezing state was recorded using the PACKWIN system (Panlab/HarvardApparatus). The percentage of freezing time was automatically recorded and the data were analyzed using Packwin 2.0 software.

Spatial working memory was assessed by recording spontaneous alternation in the Y-maze test. Mice with good working spatial memory were expected to enter a new arm of the maze without immediate re-entry to a previously visited arm. The Y-maze consists of three long, wide, and high arms 35, 10, and 15 cm (A, B, and C arms) respectively. Each mouse was placed in the center of the symmetric Y-maze and allowed to explore the three arms freely for 8 min. Any-maze (Stoelting Co., USA) was used to record the sequence and the total number of arms entered. An arm entry was defined as the entry of four limbs and the tail into one arm of the Y-maze. Nonrepetitive consecutive arm entries (i.e., ABC, BCA, or CAB) were defined as a successful triad combination. The percentage of spontaneous alterations was calculated as follows: (number of successful triad combinations)/(total number of arm entries-2) × 100. After completion of the behavioral testing, all animals were sacrificed to collect brain tissue.

### Flow cytometry analysis

Mice that had been sacrificed were perfused transcranially with ice-cold phosphate-buffered saline (PBS; pH 7.4). The hippocampus was gently placed in an MACS separation tube, the mouse brain tissue lysis procedure was selected, and two tissue dispersion treatments were performed to prepare a single-cell suspension. The cell number was measured, the cell suspension was centrifuged at 300× *g* for 10 min, and the supernatant was aspirated. The intracellular ROS levels were measured using a Reactive Oxygen Species Assay Kit (S0033S; Beyotime, Shanghai, China); 2’,7’-dichlorofluorescein-diacetate (DCFH-DA), which is easily oxidized to fluorescent dichlorofluorescein (DCF) by intracellular ROS, is the principal component. Therefore, the ROS levels were quantified. Briefly, these cells were incubated with DCFH-DA for 20 min at 37°C, then stained with 7AAD (420403; Biolegend, San Diego, CA, USA) to exclude dead cells. Finally, the cell suspension was suspended in 150 μl of PBS for subsequent testing by flow cytometry (BD Biosciences, USA).

### Measurement of SOD and MDA

Superoxide dismutase (SOD) activity levels were estimated using the Total Superoxide Dismutase Assay Kit with WST-8 (S0101; Beyotime) and the lipid peroxidation levels were estimated using the Lipid Peroxidation MDA Assay Kit (S0131S; Beyotime). The hippocampus tissues were homogenized in pre-cooled PBS for 15 min. Then, the samples were centrifuged at 12,000× *g* for 10 min at 4°C. The testing process was carried out in strict accordance with the manufacturer’s instructions.

### Western blot analysis

Hippocampus tissues were sonicated in RIPA lysis buffer (P0013B; Beyotime), and both protease and phosphatase inhibitors (Thermo Fisher, Shanghai, China) were used to prevent degradation. Protein concentrations were measured using the BCA Protein Assay Kit (P0011; Beyotime). Total protein (30 μg) was loaded into each well and samples were separated by 10% and 12.5% Tris-glycine SDS-PAGE. The proteins were transferred onto the polyvinylidene fluoride (PVDF) membranes (IPVH00010; Millipore, Shanghai, China). Then, the membranes were blocked with 5% non-fat milk for 60 min at room temperature. After blocking, the membranes were incubated with the following primary antibodies overnight at 4°C: rabbit anti-ATF4 (10835-1-AP, 1:1,000; Proteintech); rabbit anti-GADD34 (10449-1-AP, 1:1,000; Proteintech); rabbit anti-CHOP (15204-1-AP, 1:1,000; Proteintech); rabbit anti-BDNF (ab108319, 1:1,000; Abcam, Cambridge, MA, USA); rabbit anti-NMDAR2B (phospho Y1472, ab3856, 1:1,000; Abcam); rabbit anti-NMDAR2B (BS72928, 1:2,000; Bioworld); and anti-β-actin (4970, 1:20,000; Cell Signaling Technology, USA). After primary antibody incubation, the membranes were washed three times in TBST, then incubated with HRP-conjugated goat anti-rabbit IgG (BL003A, 1:20,000; Biosharp, Anhui, China) for 2 h at room temperature. Finally, the target protein bands were visualized with chemiluminescence reagents (catalog # 34580; Thermo Fisher,) and β-tubulin was used as the internal reference gene for normalization. The band intensities were measured to perform densitometric analysis using ImageJ software (version 1.53c; National Institutes of Health, Bethesda, MD, USA), the PVDF membranes were first incubated with rabbit anti-phospho-eIF2α (Ser51, BS4787, 1:1,000; Bioworld, Nanjing, China) to detect the phosphorylation of eIF2α. Then, the primary antibodies were stripped with strip buffer (21059; Thermo Scientific). After stripping, the membrane was re-blocked and incubated with rabbit anti-total eIF2α antibody (BS3651, 1:1,000; Bioworld).

### Statistical analysis

All data are presented as the mean ± SEM. Student’s t-test and analysis of variance (ANOVA) with *post-hoc* tests were used for statistical comparison, as indicated. SPSS 22.0 (IBM, Armonk, NY, USA) and GraphPad Prism software (9.0.0; GraphPad Software, CA, USA) were applied to analyze the data and graphing, respectively. A p-value < 0.05 was considered statistically significant.

## Results

### Tibial fracture surgery induced cognitive decline, accompanied by oxidative stress damage and ISR activation in the hippocampus of POCD mice

**Figure 1:**
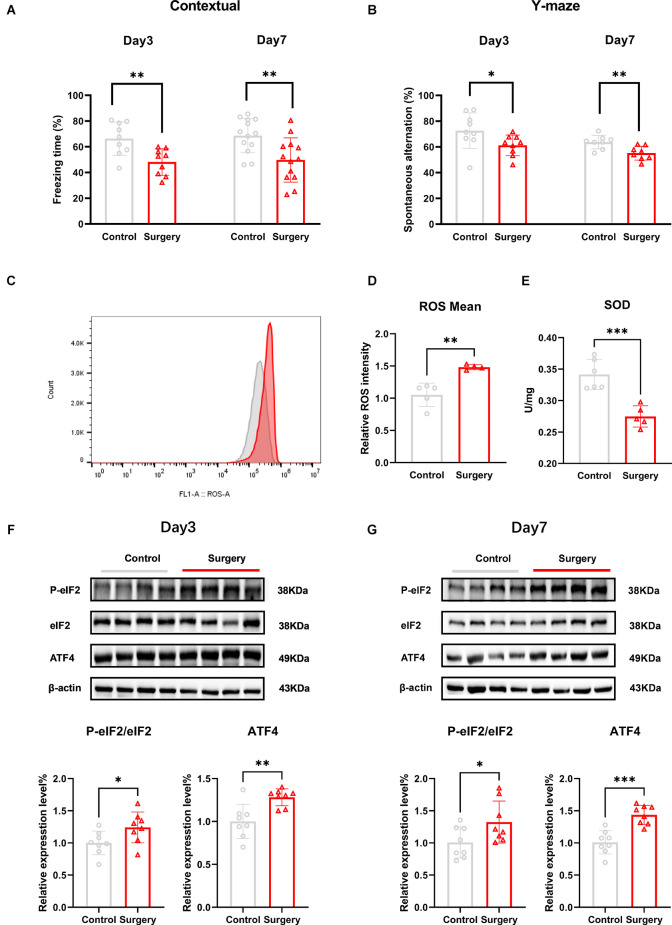
Tibial fracture surgery induces cognitive decline, accompanied by oxidative stress damage, and ISR activation in the hippocampus of POCD mice. **(A)** Freezing percentage in the contextual fear conditioning experiment on post-surgery days 3 and 7; the freezing time percentage was significantly reduced in the surgery group compared with the control group. **(B)** Spontaneous alternation was significantly decreased in the Y-maze on days 3 and 7 following tibial fracture surgery. **(C)** The average fluorescence intensity of the ROS level increased in the hippocampus of POCD mice on post-surgery day 3, in which red represents the surgery group and gray represents the control group. **(D)** Histogram quantification of ROS average fluorescence intensity of **(C)**. **(E)** The SOD level is decreased in the hippocampus of POCD mice on post-surgery day 3. **(F)** POCD increased eIF2α phosphorylation and ATF4 protein levels at post-surgery days 3 and 7 **(G)**. Data are presented as the mean ± SEM. Student’s t-test was used for statistical analysis. *p < 0.05; **p < 0.01; ***p < 0.001.

In this study, we used tibial fracture surgery to establish a POCD mice model. The fear conditioning contextual test was used to evaluate hippocampus-dependent long-term learning and memory on post-surgery days 3 and 7. The freezing time was significantly decreased in the surgery group compared to the control group on the fear conditioning context test (day 3: *t* = 3.112, *p* = 0.0067; day 7: *t* = 3.316, *p* = 0.0045; Figure 1A). No differences in freezing time were detected between groups at any time point on the FCT tone test (day 3: *t* = 0.3538, *p* = 0.7281; day 7: *t* = 1.022, *p* = 03271; Supplementary Figures S1A,B). Together, these results suggested that surgery-induced hippocampus-dependent memory declined.

To assess spatial working memory, mice were placed in a Y maze and allowed to freely explore the three arms for 8 min. Movement velocity and the total distance were not different between the two groups of mice (speed: *t* = 0.06349, *p* = 0.9495; distance: *t* = 0.02586, *p* = 0.9797; Supplementary Figure S1C,D).The percentage of spontaneous alternations was significantly lower in the surgery group compared to the control group at post-surgery days 3 and 7 (day 3: *t* = 2.138, *p* = 0.0483; day 7: *t* = 3.215, *p* = 0.0062; Figure 1B).

The levels of oxidative stress markers in the hippocampus of mice were subsequently measured. The levels of ROS were significantly higher (*t* = 4.605, *p* = 0.0025; Figures 1C,D) and SOD activity was significantly reduced (*t* = 5.215, *p* = 0.0006; Figure 1E) in the surgery group compared to the control group at post-surgery day 3.

To determine whether POCD induced changes in PeIF2α/eIF2α protein expression in POCD mice, we measured the protein levels on post-surgery days 3 and 7. Compared to the control group, the PeIF2α/eIF2α level was significantly increased in the surgery group (day 3: *t* = 2.243, *p* = 0.0416; day 7: *t* = 3.823, *p* = 0.0024; Figures 1F,G). In the ISR, P-eIF2α increased the translation of ATF4, which in turn upregulated grow arrest, DNA damage-inducible protein (GADD34), and CCAAT/enhance binding homologous protein (CHOP; Ghadge et al., 2020). The levels of ATF4 protein were also increased in the surgery group (day 3: *t* = 3.588, *p* = 0.0030; day 7: *t* = 3.112, *p* = 0.0125; Figures 1F,G). GADD34 is a regulatory subunit of the protein phosphatase PP1 that eventually dephosphorylates PeIF2α (Xu et al., 2021) and the sustained expression of CHOP induces cell apoptosis (Yi et al., 2019), while apoptosis has been reported in the hippocampus of POCD mice (Xie et al., 2021). GADD34 and CHOP protein expression was increased in the surgery group (GADD34: *t* = 2.870, *p* = 0.0131; CHOP: *t* = 3.314, *p* = 0.0051; Supplementary Figures S2A,B).

### ISRIB treatment relieved ISR in the hippocampus of POCD mice

**Figure 2:**
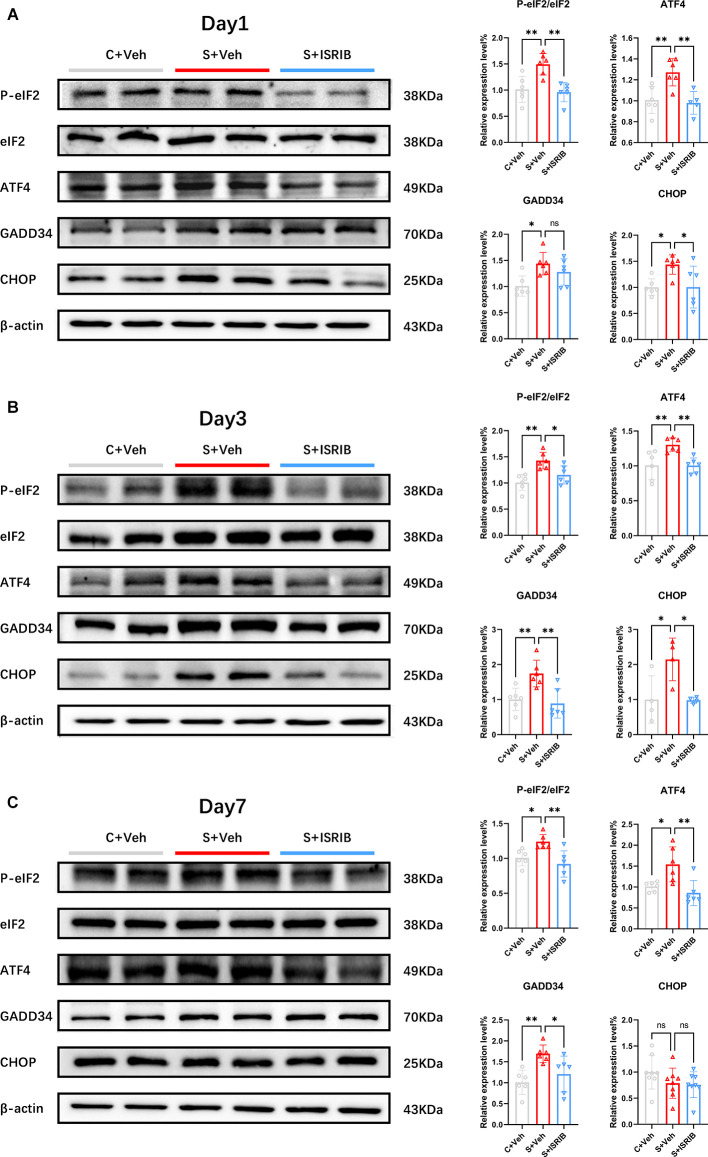
POCD induced eIF2α phosphorylation and related protein changes in the hippocampus of mice and therapy with ISRIB attenuated the changes. **(A–C)** Changes in PeIF2, eIF2, ATF4, GADD34, and CHOP protein expression on post-surgery days 1 **(A)**, 3 **(B)**, and 7 **(C)** in all groups. Relative protein expression is expressed as the ratio of the target protein to β-actin. Representative protein bands are shown on the left and quantification are shown on the right. Data are presented as the mean ± SEM. One-way ANOVA was used for statistical analysis. *p < 0.05; **p < 0.01; ns: no statistical difference.

Several studies have provided effective dosing protocols for ISRIB (Krukowski et al., 2020a,b). Considering the limitations of clinical surgical administration, we chose the single dose regimen with the same total dose (7.5 mg/kg i.p.) as the final plan. The flow diagram of the study procedure is shown in the Supplementary Figure S2C. We determined hippocampal PeIF2α/eIF2α, ATF4, GADD34, and CHOP protein expression on days 1, 3, and 7 after injecting mice intraperitoneally with ISRIB (7.5 mg/kg) post-surgery. Compared with the C + Veh group, the levels of PeIF2α/eIF2α, ATF4 protein expression were significantly increased in the S + Veh group on post-surgery days 1, 3, and 7. After the administration of ISRIB, the PeIF2α/eIF2α and ATF4 protein levels were significantly decreased in the S + ISRIB group compared with the surgery group at post-surgery days 1, 3, and 7 (Day 1 PeIF2α/eIF2α: *F*_(2,15)_ = 11.28, C + Veh vs. S + Veh: *p* = 0.0039; C + Veh vs. S + ISRIB: *p* = 0.9057; S + Veh vs. S + ISRIB: *p* = 0.0017; Day 1 ATF4: *F*_(2,14)_ = 9.814, C + Veh vs. S + Veh: *p* = 0.0061; C + Veh vs. S + ISRIB: *p* = 0.9327; S + Veh vs. S + ISRIB: *p* = 0.0043; Day 3 PeIF2α/eIF2α: *F*_(2,15)_ = 10.29, C + Veh vs. S + Veh: *p* = 0.0012; C + Veh vs. S + ISRIB: *p* = 0.2976;S + Veh vs. S + ISRIB: *p* = 0.0270; Day 3 ATF4: *F*_(2,15)_ = 8.329, C + Veh vs. S + Veh: *p* = 0.0082; C + Veh vs. S + ISRIB: *p* = 0.9995; S + Veh vs. S + ISRIB: *p* = 0.0077; Day 3 PeIF2α/eIF2α; Day 7 PeIF2α/eIF2α: *F*_(2,15)_ = 8.253, C + Veh vs. S + Veh: *p* = 0.0318; C + Veh vs. S + ISRIB: *p* = 0.5257; S + Veh vs. S + ISRIB: *p* = 0.0035; Day 7 ATF4: *F*_(2,15)_ = 8.316, C + Veh vs. S + Veh: *p* = 0.0225; C + Veh vs. S + ISRIB: *p* = 0.6622; S + Veh vs. S + ISRIB: *p* = 0.0039; Figures 2A–C).

The level of GADD34 expression increased continuously until post-surgery day 7. After ISRIB administration, there was no improvement on post-surgery day 1 between the S + Veh and S + ISRIB groups, but the level of GADD34 expression decreased on post-surgery days 3 and 7 (Day 1: *F*_(2,15)_ = 5.647, C + Veh vs. S + Veh: *p* = 0.0121; C + Veh vs. S + ISRIB: *p* = 0.1237; S + Veh vs. S + ISRIB: *p* = 0.4580; Day 3: *F*_(2,15)_ = 9.193, C + Veh vs. S + Veh: *p* = 0.0100; C + Veh vs. S + ISRIB: *p* = 0.8585; S + Veh vs. S + ISRIB: *p* = 0.0035; Day 7: *F*_(2,15)_ = 7.186, C + Veh vs. S + Veh: *p* = 0.0059; C + Veh vs. S + ISRIB: *p* = 0.5492; S + Veh vs. S + ISRIB: *p* = 0.0481; Figures 2A–C). The level of CHOP expression increased on post-surgery days 1 and 3, and ISRIB reduced the level of CHOP expression between the S + Veh and S + ISRIB groups. On post-surgery day 7, there were no differences between the three groups (Day 1: *F*_(2,15)_ = 5.093, C + Veh vs. S + Veh: *p* = 0.0362; C + Veh vs. S + ISRIB: *p* > 0.9999; S + Veh vs. S + ISRIB: *p* = 0.0366; Day 3: *F*_(2,9)_ = 6.377, C + Veh vs. S + Veh: *p* = 0.0326; C + Veh vs. S + ISRIB: *p* = 0.9985; S + Veh vs. S + ISRIB: *p* = 0.0300; Day 7: *F*_(2,21)_ = 1.682, C + Veh vs. S + Veh: *p* = 0.3223; C + Veh vs. S + ISRIB: *p* = 0.2357; S + Veh vs. S + ISRIB: *p* = 0.977).

Western blot analysis revealed that administration of ISRIB relieved the ISR in the hippocampus of POCD mice and decreased the levels of the P-eIF2α downstream protein, GADD34, and the apoptotic protein, CHOP.

### ISRIB treatment alleviated oxidative stress in the hippocampus of POCD mice

**Figure 3:**
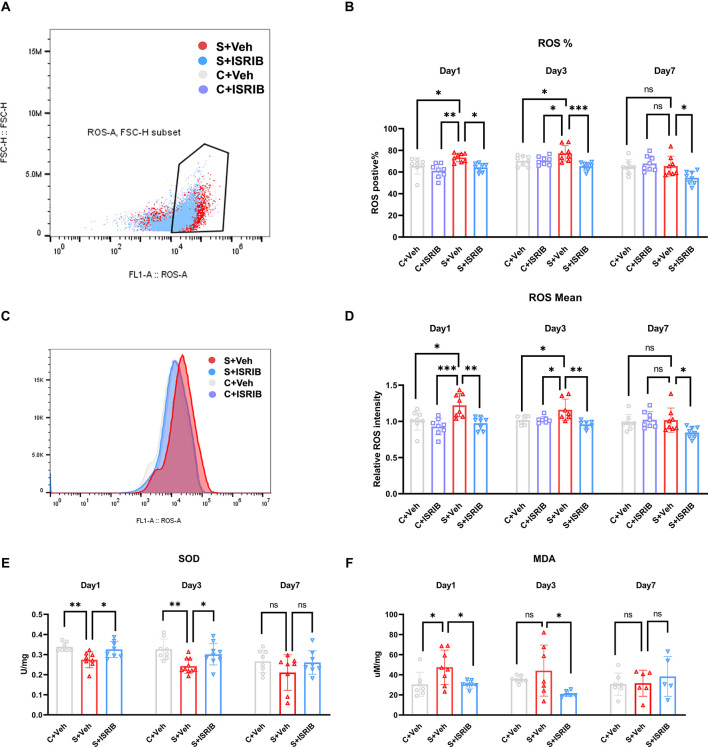
POCD induced oxidative stress in the hippocampus of mice and therapy with ISRIB attenuates the oxidative stress. **(A)** Representative ROS positive cell in flow cytometry. **(B)** The change in ROS-positive cells in the hippocampus of mice post-surgery days 1, 3, and 7 in all groups. **(C)** Representative ROS fluorescence intensity in flow cytometry. **(D)** The change in relative ROS fluorescence intensity in the hippocampus of mice post-surgery days 1, 3, and 7 in all groups. **(E)** The change in SOD levels in the hippocampus of mice post-surgery days 1, 3, and 7 in all groups. **(F)** The change in MDA levels in the hippocampus of mice post-surgery days 1, 3, and 7 in all groups. Data are presented as the mean ± SEM. One-way ANOVA was used for statistical analysis. *p < 0.05; **p < 0.01; ***p < 0.001; ns: no statistical difference.

To determine oxidative stress in POCD mice hippocampus, we used flow cytometry to evaluate ROS fluorescence intensity and determine the percentage of ROS-positive cells. Then, we evaluated the relative SOD and MDA levels. At post-surgery days 1 and 3, the percentage of ROS-positive cells in the hippocampus of POCD mice was significantly higher in the S + Veh group compared to the C + Veh group, and after ISRIB treatment the percentage of ROS-positive cells decreased in the S + ISRIB group compares to the S + Veh group (Day 1: *F*_(3,28)_ = 6.713, C + Veh vs. S + Veh: *p* = 0.0495; C + ISRIB vs. S + Veh: *p* = 0.0010; S + ISRIB vs. S + Veh: *p* = 0.0156; Day 3: *F*_(3,27)_ = 7.726, C + Veh vs. S + Veh: *p* = 0.0441; C + ISRIB vs. S + Veh: *p* = 0.0288; S + ISRIB vs. S + Veh: *p* = 0.0003; Figures 3A,B). At post-surgery day 7, there was no difference between the C + Veh group and the S + Veh group; the percentage of ROS-positive cells in the hippocampus in the S + ISRIB group was decreased compared to the S + Veh group (Day 7: *F*_(3,26)_ = 5.038, S + ISRIB vs. S + Vehp = 0.0221; Figure 3B). At post-surgery days 1 and 3, the relative ROS fluorescence intensity in the hippocampus of POCD mice was significantly higher in the S + Veh group compared to the C + Veh group. Following ISRIB treatment, the relative ROS fluorescence intensity was decreased in the S + ISRIB group compared to the S + Veh group (Day 1: *F*_(3,28)_ = 8.486, C + Veh vs. S + Veh: *p* = 0.0131; C + ISRIB vs. S + Veh: *p* = 0.0004; S + ISRIB vs. S + Veh: *p* = 0.0027; Day 3: *F*_(3,24)_ = 6.769, C + Veh vs. S + Veh: *p* = 0.0250; C + ISRIB vs. S + Veh: *p* = 0.0334; S + ISRIB vs. S + Veh: *p* = 0.0012; Figures 3C,D). At post-surgery day 7, there was no difference between the C + Veh group and the S + Veh group; the relative ROS fluorescence intensity in the hippocampus in the S + ISRIB group was decreased compared to the S + Veh group (Day 7: *F*_(3,26)_ = 3.719, S + ISRIB vs. S + Veh: *p* = 0.0321; Figure 3D). We also determined the percentage of ROS-positive cells and ROS fluorescence intensity in the prefrontal cortex (Supplementary Figures S3A,B). The changes were the same as the changes in the hippocampus, but the oxidative stress levels in the prefrontal cortex persisted until post-surgery day 7.

We then analyzed the oxidative stress-related SOD and MDA activities. The results showed that SOD activity was significantly reduced in the S + Veh group compared to the C + Veh group on post-surgery days 1 and 3. The SOD activity was increased in the S + ISRIB group compared to the S + Veh group (Day 1: *F*_(2,20)_ = 7.136, C + Veh vs. S + Veh: *p* = 0.0058; S + ISRIB vs. S + Veh: *p* = 0.0229; Day 3: *F*_(2,25)_ = 7.576, C + Veh vs. S + Veh: *p* = 0.0028; S + ISRIB vs. S + Veh: *p* = 0.027; Figure 3E). At post-surgery day 7, the SOD activity did not differ between groups (Day 7: *F*_(2,21)_ = 1.559, C + Veh vs. S + Veh: *p* = 0.2675; S + ISRIB vs. S + Veh: *p* = 0.3360; Figure 3E). The MDA level was increased in the S + Veh group compared to the C + Veh group at post-surgery day 1, while administration of ISRIB decreased the MDA level in the S + ISRIB group compared to the S + Veh group (Day 1: *F*_(2,19)_ = 4.572, C + Veh vs. S + Veh: *p* = 0.0432; S + ISRIB vs. S + Veh: *p* = 0.0475; Figure 3F). At post-surgery day 3, there was no difference between the C + Veh and S + Veh groups, but the MDA level was decreased in the S + ISRIB group compared to the C + Veh group (Day 3: *F*_(2,17)_ = 3.696, C + Veh vs. S + Veh: *p* = 0.5669; S + ISRIB vs. S + Veh: *p* = 0.0382; Figure 3F); at post-surgery day 7, there was no difference between the groups (Day 7: *F*_(2,15)_ = 0.4600, C + Veh vs. S + Veh: *p* = 0.9932; S + ISRIB vs. S + Veh: *p* = 0.7247; Figure 3F).

### ISRIB treatment mitigated cognition dysfunction of POCD mice

**Figure 4:**
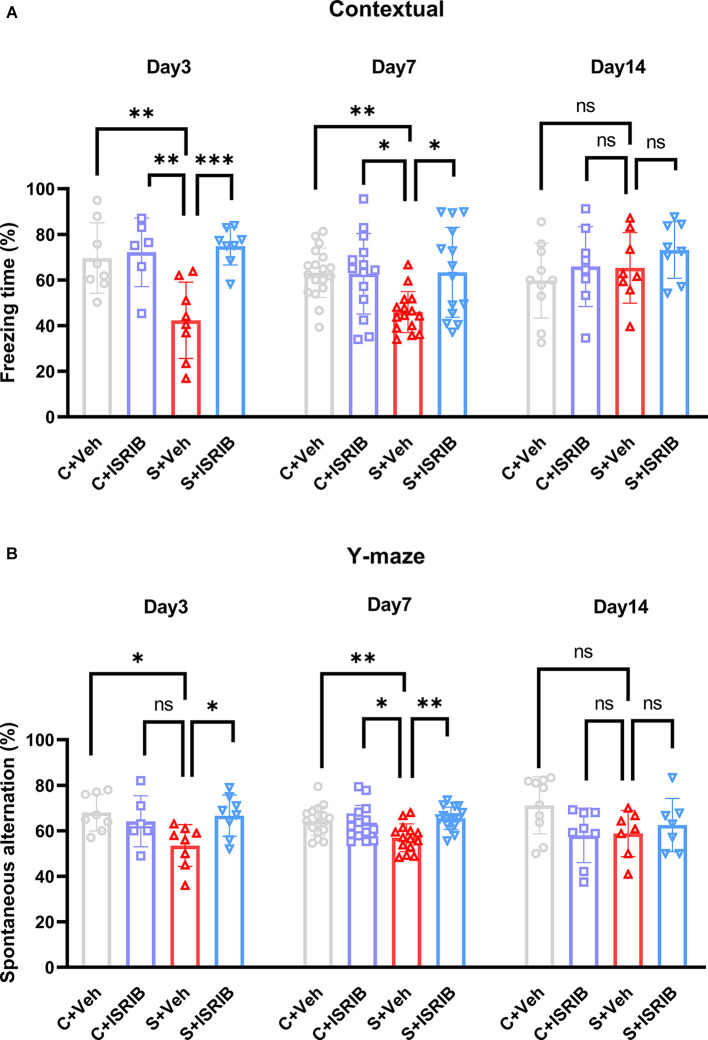
Effect of ISRIB pretreatment on cognitive performance in POCD mice. **(A)** Freezing time in the context test of FCT. A significantly higher freezing time was observed in the S + ISRIB group compared to the S + vehicle group on post-surgery days 3 and 7. No differences in freezing time were observed on post-surgery day 14 among the groups. **(B)** Spontaneous alternation was rescued by ISRIB on post-surgery days 3 and 7; there were no differences in spontaneous alternation on post-surgery day 14. Data are presented as the mean ± SEM. One-way ANOVA was used for statistical analysis. *p < 0.05; **p < 0.01; ***p < 0.001; ns: no statistical difference.

We next determined if pharmacologic inhibition of the ISR could modify the POCD-induced spatial learning deficits. We injected mice intraperitoneally with ISRIB (7.5 mg/kg) after tibial fracture surgery. The freezing time was significantly decreased in the S + Veh group compared to the C + Veh group in the FCT context test, while administration of ISRIB significantly increased the freezing time in the S + Veh group (Day 3: *F*_(3,26)_ = 8.833, C + Veh vs. S + Veh: *p* = 0.0037; C + ISRIB vs. S + Veh: *p* = 0.0033; S + ISRIB vs. S + Veh: *p* = 0.0006; Day 7: *F*_(3,57)_ = 5.200, C + Veh vs. S + Veh: *p* = 0.0071; C + ISRIB vs. S + Veh: *p* = 0.0159; S + ISRIB vs. S + Veh: *p* = 0.0116; Day 14: *F*_(3,30)_ = 1.084, C + Veh vs. S + Veh: *p* = 0.8757; C + ISRIB vs. S + Veh: *p* = 0.9999; S + ISRIB vs. S + Veh: *p* = 0.7520; Figure 4A). No differences in freezing time were detected between the groups in the FCT tone test (Day 3: *F*_(3,26)_ = 0.4910, C + Veh vs. S + Veh: *p* = 0.6862; C + ISRIB vs. S + Veh: *p* = 0.9790; S + ISRIB vs. S + Veh: *p* = 0.8012; Day 7: *F*_(3,26)_ = 1.240; C + Veh vs. S + Veh: *p* = 0.4586; C + ISRIB vs. S + Veh: *p* = 0.7901; S + ISRIB vs. S + Veh: *p* = 0.9981; Day 14: *F*_(3,30)_ = 0.5093, C + Veh vs. S + Veh: *p* = 0.8950; C + ISRIB vs. S + Veh: *p* = 0.6364; S + ISRIB vs. S + Veh: *p* = 0.8163; Supplementary Figure S4A). The mice in the S + Veh group exhibited fewer spontaneous alterations than the mice in C + Veh group in the Y-maze test, which was reversed by ISRIB treatment (Day 3: *F*_(3,26)_ = 3.965, C + Veh vs. S + Veh: *p* = 0.0220; C + ISRIB vs. S + Veh: *p* = 0.1742; S + ISRIB vs. S + Veh: *p* = 0.0429; Day 7: *F*_(3,58)_ = 5.468, C + Veh vs. S + Veh: *p* = 0.0099; C + ISRIB vs. S + Veh: *p* = 0.0308; S + ISRIB vs. S + Veh: *p* = 0.0024; Day 14: *F*_(3,28)_ = 2.400, C + Veh vs. S + Veh: *p* = 0.1652; C + ISRIB vs. S + Veh: *p* = 0.9991; S + ISRIB vs. S + Veh: *p* = 0.9315; Figure 4B).

These findings are consistent with the previous studies that ISRIB acted downstream of P-eIF2α to restore memory and indicated that activated ISR in the hippocampus of POCD mice may be the basis for cognition dysfunction.

**Figure 5:**
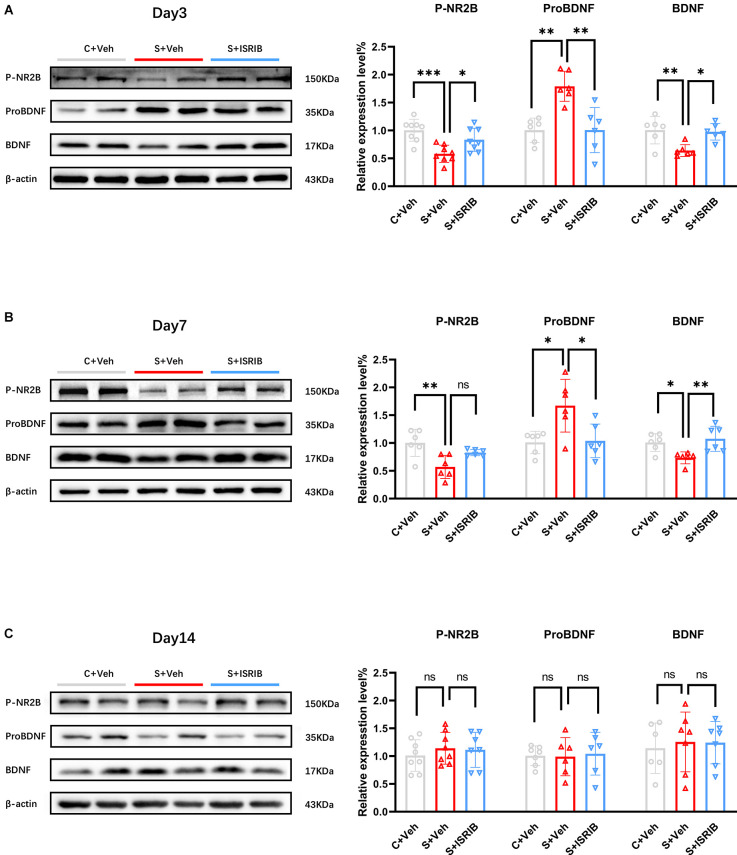
Effect of ISRIB pretreatment on levels of P-NR2B, ProBDNF, and BDNF protein expression in POCD mice. **(A)** Changes in P-NR2B, ProBDNF, and BDNF protein expression at post-surgery day 3 in all groups. Representative protein bands are shown on the left and quantification are shown on the right. **(B)** Changes in P-NR2B, ProBDNF, and BDNF protein expression at post-surgery day 7 in all groups. **(C)** Changes in P-NR2B, ProBDNF, and BDNF protein expression at post-surgery day 14 in all groups. One-way ANOVA was used for statistical analysis. *p < 0.05; **p < 0.01; ***p < 0.001; ns: no statistical difference.

We first found that there was no difference in the expression level of NR2B total protein in the hippocampus of POCD mice at post-surgery day 3, 7, and 14 (Day 3, *F*_(2,15)_ = 0.02408, C + Veh vs. S + Veh: *p* = 0.9973, S + ISRIB vs. S + Veh: *p* = 0.9748; Day 7, *F*_(2,15)_ = 0.03400, C + Veh vs. S + Veh: *p* = 0.9969, S + ISRIB vs. S + Veh: *p* = 0.9652; Day14, *F*_(2,15)_ = 0.1328, C + Veh vs. S + Veh: *p* = 0.9255, S + ISRIB vs. S + Veh: *p* = 0.8755; Supplementary Figure S5A). POCD mice had decreased hippocampus P-NR2B and BDNF protein levels at post-surgery days 1, 3, and 7, while the pro BDNF level was increased at post-surgery days 1, 3, and 7 (Day 1, P-NR2B: *F*_(2,15)_ = 9.987, C + Veh vs. S + Veh: *p* = 0.0081, S + ISRIB vs. S + Veh: *p* = 0.8125; ProBDNF: *F*_(2,9)_ = 9.245, C + Veh vs. S + Veh: *p* = 0.0082, S + ISRIB vs. S + Veh: *p* = 0.0191; BDNF: *F*_(2,9)_ = 12.18, C + Veh vs. S + Veh: *p* = 0.0127, S + ISRIB vs. S + Veh: *p* = 0.0029; Supplementary Figure S5B). Administration of ISRIB increased the P-NR2B and BDNF levels on post-surgery days 3 and 7, while decreasing the pro BDNF level at the same time (Day 3, P-NR2B: *F*_(2,21)_ = 10.22, C + Veh vs. S + Veh: *p* = 0.0006, S + ISRIB vs. S + Veh: *p* = 0.0359; Pro BDNF: *F*_(2,15)_ = 13.07, C + Veh vs. S + Veh: *p* = 0.0013, S + ISRIB vs. S + Veh: *p* = 0.0014; BDNF: *F*_(2,15)_ = 7.910, C + Veh vs. S + Veh: *p* = 0.0072, S + ISRIB vs. S + Veh: *p* = 0.0131; Day7, P-NR2B: *F*_(2,15)_ = 8.090, C + Veh vs. S + Veh: *p* = 0.0031, S + ISRIB vs. S + Veh: *p* = 0.0739; ProBDNF: *F*_(2,15)_ = 7.026, C + Veh vs. S + Veh: *p* = 0.0124, S + ISRIB vs. S + Veh: *p* = 0.0163; BDNF: *F*_(2,15)_ = 6.793, C + Veh vs. S + Veh: *p* = 0.0343, S + ISRIB vs. S + Veh: *p* = 0.0089; Figures 5A,B). At post-surgery day 14, the p-NR2B, BDNF, and ProBDNF levels did not differ between the groups (Day 14, P-NR2B: *F*_(2,20)_ = 0.4260, C + Veh vs. S + Veh: *p* = 0.6597, S + ISRIB vs. S + Veh: *p* = 0.9830; ProBDNF: *F*_(2,18)_ = 0.1260, C + Veh vs. S + Veh: *p* = 0.9901, S + ISRIB vs. S + Veh: *p* = 0.9337; BDNF: *F*_(2,20)_ = 0.3681, C + Veh vs. S + Veh: *p* = 0.6792, S + ISRIB vs. S + Veh: *p* = 0.8568; Figure 5C).

## Discussion

In this study, we used tibial fracture surgery to establish a POCD mouse model. The hippocampus is the key region of the brain involved in learning and memory. We observed significant oxidative stress damage and ISR activation in the hippocampus of POCD mice. A novel small molecule, ISRIB, reset the ISR and relieved oxidative stress damage in the hippocampus of POCD mice. More importantly, treatment with ISRIB ameliorated cognitive dysfunction in POCD mice.

Abundant evidence has revealed the role of oxidative stress damage in the pathophysiology underlying neurodegenerative diseases (Radi et al., 2014; Sumien et al., 2021). Confounding multi-factorial causes of POCD and oxidative stress are important in the pathogenesis of POCD (Netto et al., 2018). Our research group used natural extracts, such as green tea polyphenols (Song et al., 2019) and grape seed procyanidin (Gong et al., 2020), to alleviate oxidative stress levels in the hippocampus, both of which reversed the cognitive dysfunction induced by long-term isoflurane anesthesia. How to alleviate the level of oxidative stress caused by surgery may be one of the strategies for the treatment of POCD. In this study, we detected the following common indicators of oxidative stress: ROS, SOD’, and malondialdehyde (MDA). In agreement with a previous study, we demonstrated decreased SOD activity and increased ROS and MDA expression in the hippocampus of the surgery group (Xie et al., 2021; Figure 3). We confirmed that there was oxidative stress damage in the hippocampus of POCD mice. Many factors mediate the occurrence of oxidative stress damage and mitochondria are closely related to oxidative stress (Islam, 2017). Recent studies have shown that ISR regulates the level of oxidative stress through mitochondria (Guo et al., 2020). For example, ISR selectively targets mitochondrial complex components for translation inhibition and reduces complex-associated ROS production (Zhang et al., 2021). Excessive activation of ISR will also lead to mitochondrial energy imbalance and respiratory chain dysfunction (Kaspar et al., 2021).

The ISR responds to a variety of different stress conditions to maintain cell homeostasis (Harding et al., 2003). ISR operates the last step of gene expression (reprogramming translation). Protein translation initiation requires the ternary complex to transfer GTP energy (Hinnebusch et al., 2016); eIF2α is a part of the ternary complex and can help provide the energy required for protein synthesis initiation to exchange GTP for GDP (Algire et al., 2005). The eIF2B is a guanylate exchange factor that can help GDP dissociate from the ternary complex. The conversion process from GDP-to-GTP is a speed-limiting step requiring eIF2B catalysis (Wortham et al., 2014). When cells face various pressures, four special kinases phosphorylate eIF2α to P-eIF2α, which inhibit the binding of the ternary complex to GTP, thus decreasing global protein synthesis and inducing the translation of specific genes, including the transcription factor, ATF4 (Pakos-Zebrucka et al., 2016). The most important element of ISR signaling is eIF2α phosphorylation and increased expression of ATF4. The increase in P-eIF2α and ATF4 has been observed in many neurodegenerative diseases (Oliveira and Lourenco, 2016; Zhu et al., 2019; Ghadge et al., 2020; Chang et al., 2022; Hu et al., 2022). Our results also showed that the same result occurs in POCD mice. We noted memory impairment of mice on post-surgery days 3 and 7, and found that P-eIF2α and ATF protein expression was increased (i.e., ISR activation) in the hippocampus of mice at the same time. The persistent activation of ISR and ATF4 translation induces transcription of the proapoptotic transcription factors, CHOP and GADD34 (Zhang and Kaufman, 2008; Yi et al., 2019). CHOP arrests the cell cycle and induces cell death. We observed that the increased CHOP gene activity persisted until the 3rd day after surgery, which may provide an explanation for apoptosis in the hippocampus of POCD mice (Wang et al., 2018, 2022). GADD34, a regulatory subunit of the protein phosphatase, PP1, is induced as a consequence of increased P-eIF2α abundance (Xu et al., 2021). We also obtained the same results on post-surgery days 1 and 3; GADD34 protein expression was increased in the surgery group. On post-surgery day 7, GADD34 continued to exhibit an increasing trend in the surgery group.

To further explore whether ISR activation intervention improved the cognitive impairment in POCD mice caused by oxidative stress damage, we used an ISRIB, which was first demonstrated to be an ideal ISR inhibitor that blocks the downstream effect of P-eIF2α (Sidrauski et al., 2013). ISRIB combines guanine nucleotide exchange factor (eIF2B) to help restore protein translation so that eIF2B will not inhibit the ISR response when ISR is strongly activated (Zyryanova et al., 2018). ISRIB has good pharmacokinetic properties and brain penetration (Sidrauski et al., 2015), with no toxicity, even at saturating concentrations (Rabouw et al., 2019). Multiple dose injections of ISRIB attenuate the phosphorylation of eIF2α and reset ISR (Oliveira et al., 2021). Due to the time constraints of clinical operation, we chose the single dose regimen (7.5 mg/kg) with the same total dose as previously reported (Chou et al., 2017; Krukowski et al., 2020a). Administration of ISRIB alleviated ISR in the hippocampus of POCD mice (Figure 2). PeIF2α, ATF4, and GADD34 protein expression increased in the surgery group at post-surgery days 1, 3, and 7, while the CHOP level increased until post-surgery day 3. After treatment with ISRIB, the expression of the genes related to ISR activation was reduced compared to the surgery group. In addition and more importantly, ISRIB attenuated the oxidative stress levels in the hippocampus of POCD mice, indicating that the neuroprotective effects of ISRIB may correlate with the antioxidant effects. At the same time, we also measured the ROS level in the prefrontal cortex of POCD mice and found that ROS increased and lasted until post-surgery day 7 (Supplementary Figure S3). Administration of ISRIB also reduced the ROS levels in the prefrontal cortex of POCD mice.

The most important feature of POCD is the impairment of cognitive function, which manifested as a decline in spatial memory. To assess cognitive impairment in mice, we chose FCT to detect hippocampus-dependent spatial memory and the Y-maze to detect spatial working memory. We observed that the POCD mice had cognitive memory impairment on post-surgery days 3 and 7, which manifested as a decrease in the percentage of FCT freezing time and a decrease in the percentage of spontaneous alternations in the Y-maze (Figure 4). In agreement with our previous results, we did not observe a difference at this stage of FCT tone test (Supplementary Figure S4), which may be because conditional fear memory is also closely related to the basolateral amygdale (Tronson et al., 2012). Previous studies have indicated that continuous activation of ISR affects long-term memory formation (Hu et al., 2022) and ISRIB can reverse age-related memory decline in mice (Krukowski et al., 2020a). In our study, the cognitive impairment of mice was successfully restored by administration of ISRIB, but no difference was observed on post-surgery day 14. Taken together, our data suggested that surgery induced cognitive impairment, and the impairment could be attenuated by ISRIB. To elucidate the molecular mechanism underlying cognitive impairment, our previous studies showed that two cognitive-related proteins, P-NR2B and BDNF, are damaged in the hippocampus of POCD mice (Song et al., 2019; Gong et al., 2020). In the current experiments, we also detected these two proteins. NMDAR, a class of ionotropic glutamate receptors, is known for its role in the induction of long-term potentiation (LTP) and learning and memory (Luscher and Malenka, 2012). Decreased phosphorylation of the NMDAR subunit, NR2B, will cause impairment of Ca^2+^ influx and affect the formation of LTP (Wang et al., 2017). Brain-derived neurotrophic factor (BDNF) belongs to the neurotrophic family. BDNF regulates synaptic transmission and LTP, thus playing a role in the formation of certain types of memory (Leal et al., 2014). BDNF exists in precursor and mature forms. A recent study suggested that an imbalance in precursor and mature BDNF causes cognitive decline in POCD mice (Xue et al., 2022), which is consistent with the findings in the current study. Our results revealed that downregulation of P-NR2B and BDNF in the hippocampus of POCD mice was in synchrony with cognition impairment. Decreased BDNF and increased proBDNF expression were reversed after treatment with ISRIB post-surgery days 1, 3, and 7. On post-surgery day 14, there was no difference in BDNF and pro BDNF expression between the groups. In addition, ISRIB administration recovered P-NR2B protein expression post-surgery days 1 and 3. At post-surgery day 7, P-NR2B still exhibited a recovery trend. These outcomes demonstrated the possible molecular mechanism underlying ISRIB attenuation of POCD-induced cognition impairment. In addition to oxidative stress, there are many other pathogenic mechanisms of POCD. For example, the damage of the blood-brain barrier in POCD is one of the causes of cognitive impairment (Zhang et al., 2016; Geng et al., 2018). However, we only know that ISRIB can freely penetrate the blood-brain barrier (Sidrauski et al., 2013; Krukowski et al., 2020a), but it is not clear whether ISRIB has a protective effect on the blood-brain barrier.

Our findings will provide a preventive strategy by decreasing P-eIF2α for POCD caused by oxidative stress damage; however, we still need further investigation to further delineate the precise mechanism.

## Conclusions

In conclusion, we have provided evidence that the ISR was associated with oxidative stress inducing POCD. Pharmacological attenuation of the ISR by ISRIB decreased oxidative stress damage in the hippocampus of POCD mice and mitigated cognitive dysfunction.

## Data Availability Statement

The raw data supporting the conclusions of this article will be made available by the authors, without undue reservation.

## Ethics Statement

The animal study was reviewed and approved by the Ethics Committee (license no. 2020AE01110) of the Affiliated Drum Tower Hospital (Medical School of Nanjing University, Nanjing, China).

## Authors Contributions

LJ and RD designed and conducted the experiments, analyzed and interpreted the data, and drafted the manuscript. MX and YL assisted with behavioral test and tissue processing. JX assisted with Flow cytometry and analyzed the data. ZM, TX, and XG participated in study design and coordination, secured funding for the project and critically revised the manuscript. All authors contributed to the article and approved the submitted version.

## Funding

This study was supported by grants from the National Natural Science Foundation of China (Grant numbers: 81730033, 82171193), the Key Talent Project for Strengthening Health during the 13th Five-Year Plan Period (Grant number: ZDRCA2016069), and the National Key R&D Program of China (Grant number: 2018YFC2001901).

## Supplementary Material

Supplementary Figure S1 **(A)** In the tone test of FCT, no differences in freezing time were detected between groups on post-surgery days 3 and 7 **(B)**. **(C)** Movement velocity and total distance **(D)** were not different between the two groups of mice, excluding defects in motor ability. Data are presented as the mean ± SEM. Student’s t-test was used for statistical analysis. **p* < 0.05; ***p* < 0.01; ****p* < 0.001; ns: no statistical difference.

Supplementary Figure S2 **(A)** Representative bands shown for GADD34 and CHOP at post-surgery day 3 between the control and surgery groups. **(B)** Quantification of GADD34 and CHOP protein expression is presented on post-surgery day 3. Data are presented as the mean ± SEM. Student’s t-test was used for statistical analysis. **p* < 0.05; ***p* < 0.01; ****p* < 0.001.

Supplementary Figure S3 **(A)** The change in ROS-positive cells in the prefrontal cortex of POCD mice post-surgery days 1, 3, and 7 in all groups. **(B)** The changes in relative ROS fluorescence intensity in the prefrontal cortex of POCD mice post-surgery days 1, 3, and 7 in all groups. Data are presented as the mean ± SEM. One-way ANOVA was used for statistical analysis. **p* < 0.05; ***p* < 0.01; ****p* < 0.001; ns: no statistical difference.

Supplementary Figure S4 **(A)** In the FCT tone test, no differences in freezing time were detected between groups on post-surgery days 3, 7, and 14. Data are presented as the mean ± SEM. One-way ANOVA was used for statistical analysis. **p* < 0.05; ***p* < 0.01; ****p* < 0.001; ns: no statistical difference.

Supplementary Figure S5 **(A)** Representative bands are shown for P-NR2B, BDNF, and proBDNF on post-surgery day 1 between the three groups. **(B)** The quantification of P-NR2B, BDNF, and proBDNF protein expression are presented on post-surgery day 3. Data are presented as the mean ± SEM. One-way ANOVA was used for statistical analysis. **p* < 0.05; ***p* < 0.01; ****p* < 0.001; ns: no statistical difference.
