# Supplementary material for: Inhibition of the integrated stress response reverses oxidative stress damage-induced postoperative cognitive dysfunction
Source: Front Cell Neurosci. 2022 Sep 21;16:992869. doi: 10.3389/fncel.2022.992869 (PMC9534309; doi:10.3389/fncel.2022.992869)
Supplement: Supplementary Figure S1 — (A) In the tone test of FCT, no differences in freezing time were detected between groups on post-surgery days 3 and 7 (B). (C) Movement velocity and total distance (D) were not different between the two groups of mice, excluding defects in motor ability. Data are presented as the mean ± SEM. Student’s t-test was used for statistical analysis. *p < 0.05; **p < 0.01; ***p < 0.001; ns: no statistical difference. [file Data_Sheet_1.docx]

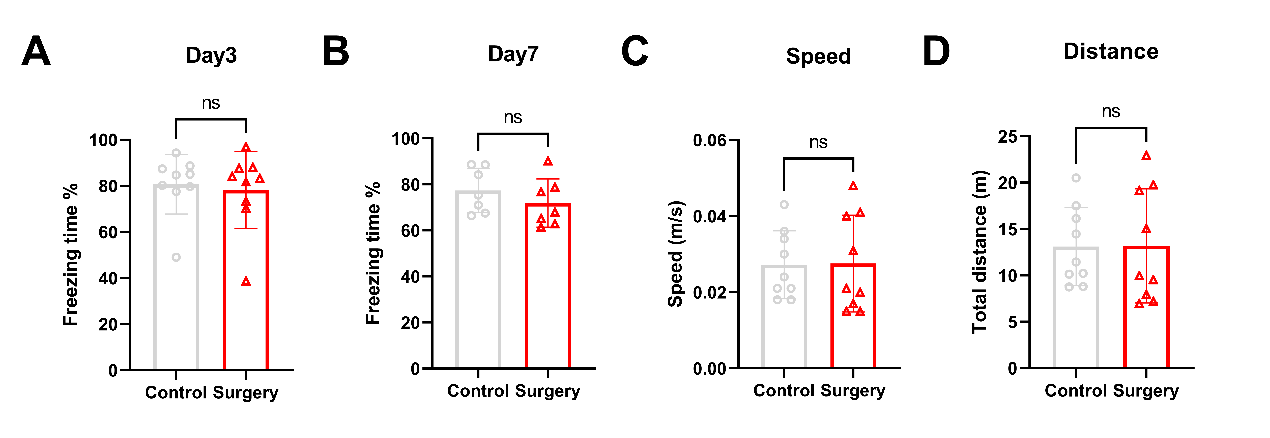


**Supplemental Figure 1.**

(A) In the tone test of FCT, no differences in freezing time were detected between groups at post-surgery days 3 and 7 (B) (Day3:n=9,Day7:n=7). (C) Movement velocity and total distance (n=9) (D) were not different between the two groups of mice, excluding defects in motor ability (n=9). Data are presented as the mean ±SEM. Student’s t-test was used for statistical analysis. *p<0.05; **p<0.01; ***p<0.001.


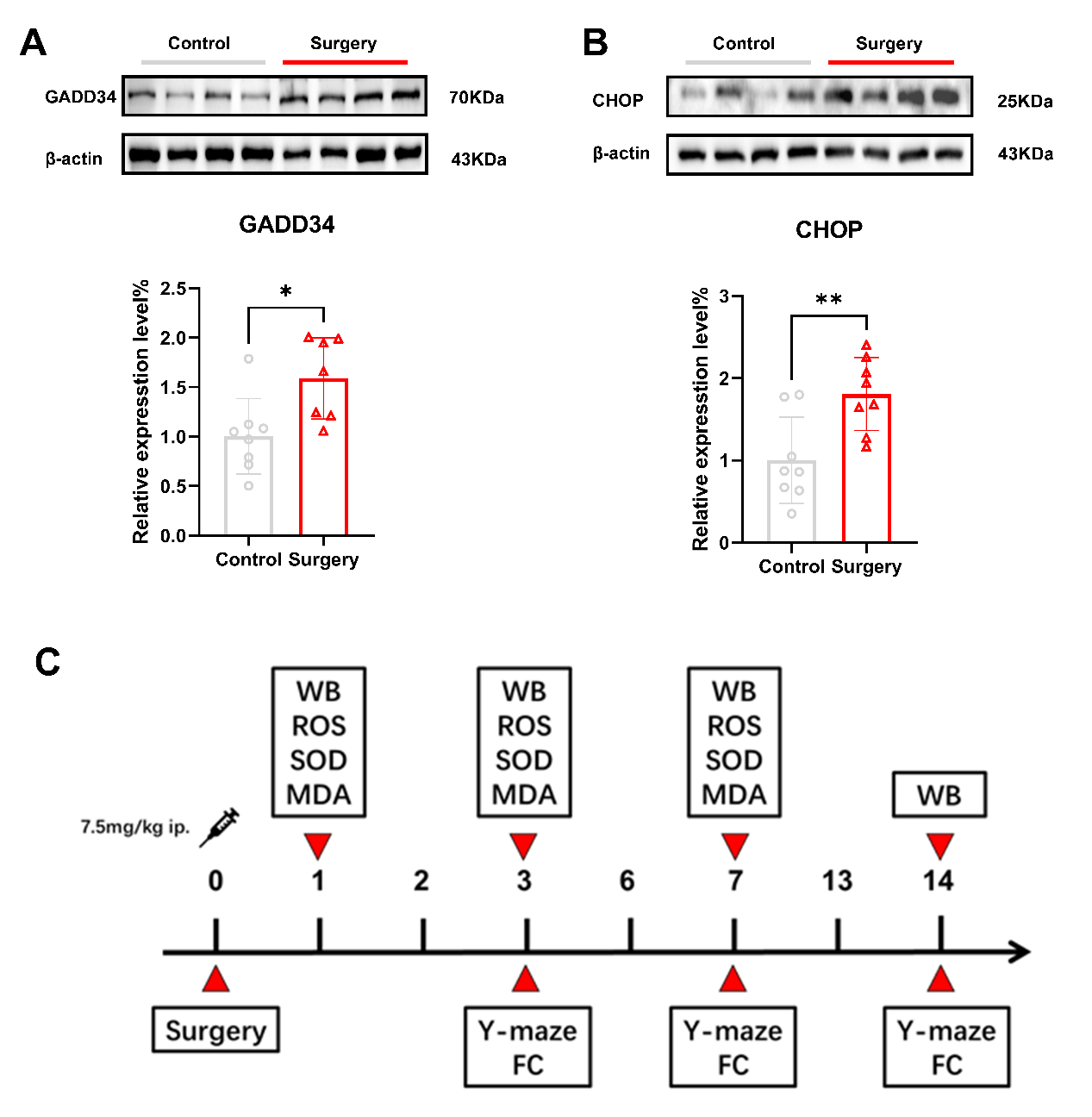


**Supplemental Figure 2.**

(A) Representative bands and quantification of GADD34 at post-surgery day 3 between the control and surgery groups (Control:n=8,Surgery:n=7). (B) Representative bands and quantification of CHOP at post-surgery day 3 between the control and surgery groups (n=8). Data are presented as the mean ±SEM. Student’s t-test was used for statistical analysis. *p<0.05; **p<0.01; ***p<0.001. (C) Flow chart of the experiment. WB, Western blot; FC, fear conditioning


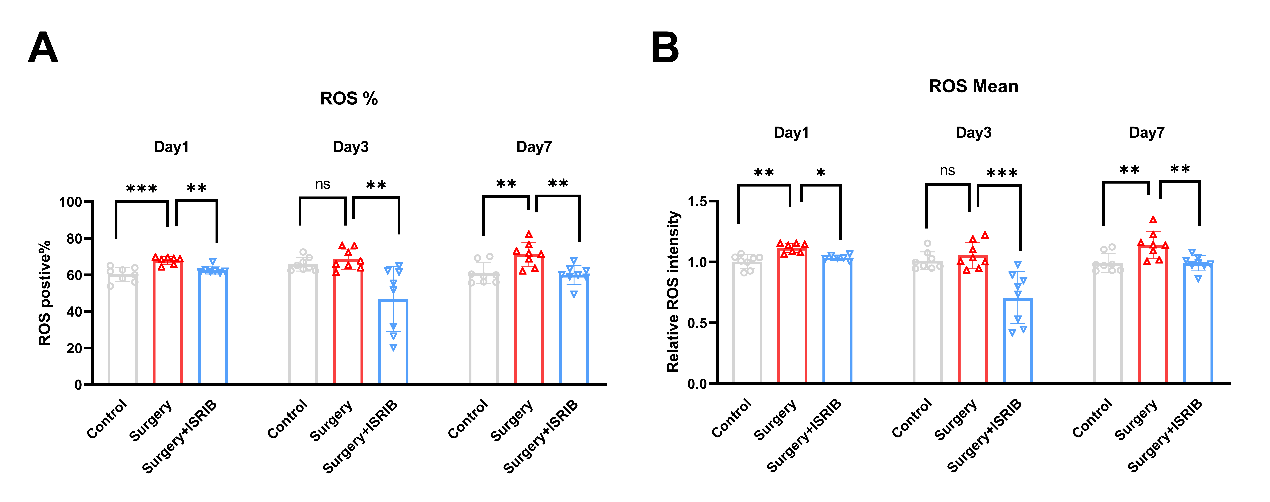


**Supplemental Figure 3.**

(A) The change in ROS-positive cells in the prefrontal cortex of POCD mice post-surgery days 1, 3, and 7 in all groups(Day1:Control:n=8,Surgery:n=7,Surgery+ISRIB:n=7;Day3: n=8;Day7:n=8). (B) The changes in relative ROS fluorescence intensity in the prefrontal cortex of POCD mice post-surgery days 1, 3, and 7 in all groups(Day1:Control:n=8,Surgery:n=7,Surgery+ISRIB:n=6;Day3: n=8;Day7:n=8). Data are presented as the mean ± SEM. One-way ANOVA was used for statistical analysis. *p<0.05; **p<0.01; ***p<0.001.


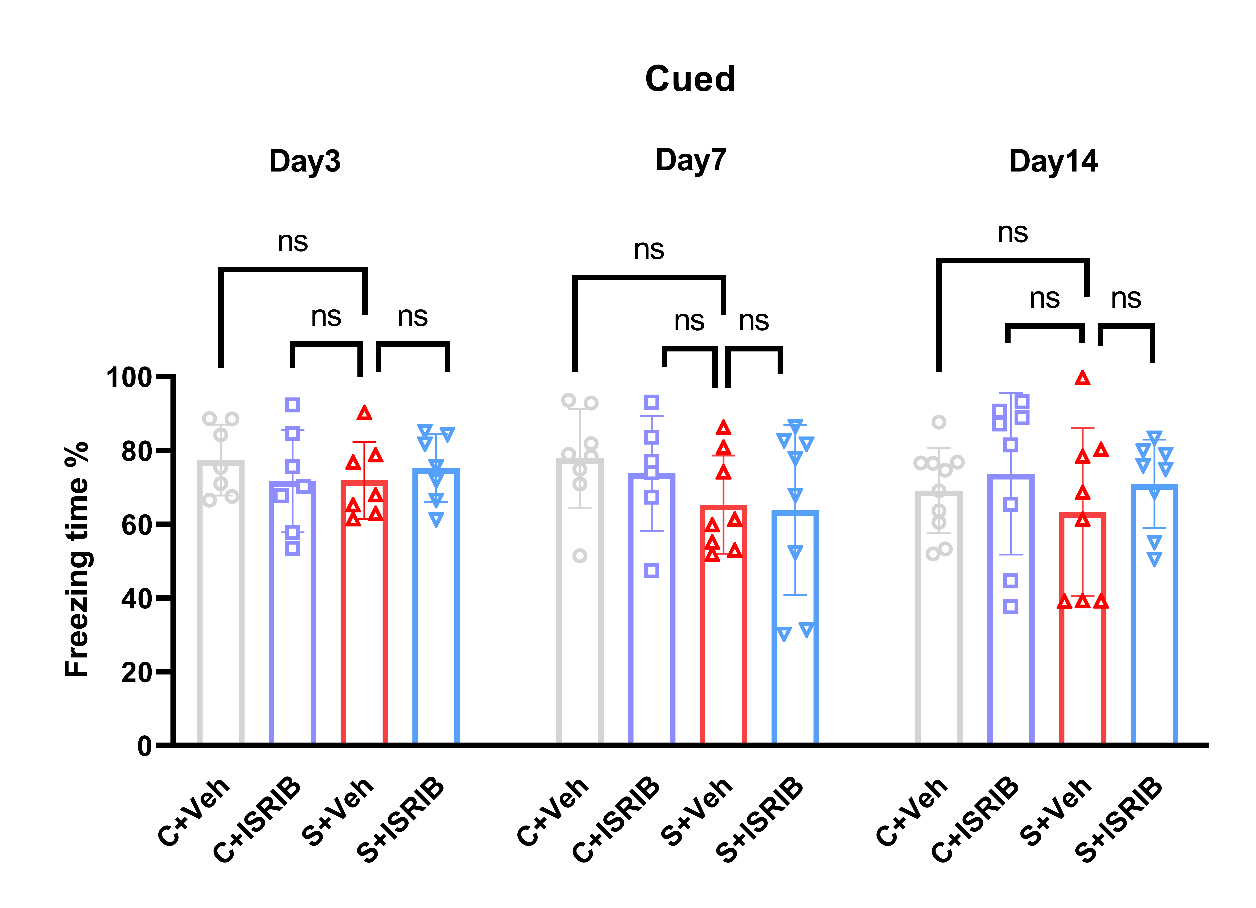


**Supplemental Figure 4.**

(A) In the FCT tone test, no differences in freezing time were detected between groups at post-surgery days 3, 7, and 14 (Day3:n=7;Day7:C+Veh:n=8,C+ISRIB:n=6,S+Veh:n=8,S+ISRIB:n=8;Day14:C+Veh:n=10,C+ISRIB:n=8,S+Veh:n=8,S+ISRIB:n=8). Data are presented as the mean ± SEM. One-way ANOVA was used for statistical analysis. *p<0.05; **p<0.01; ***p<0.001.


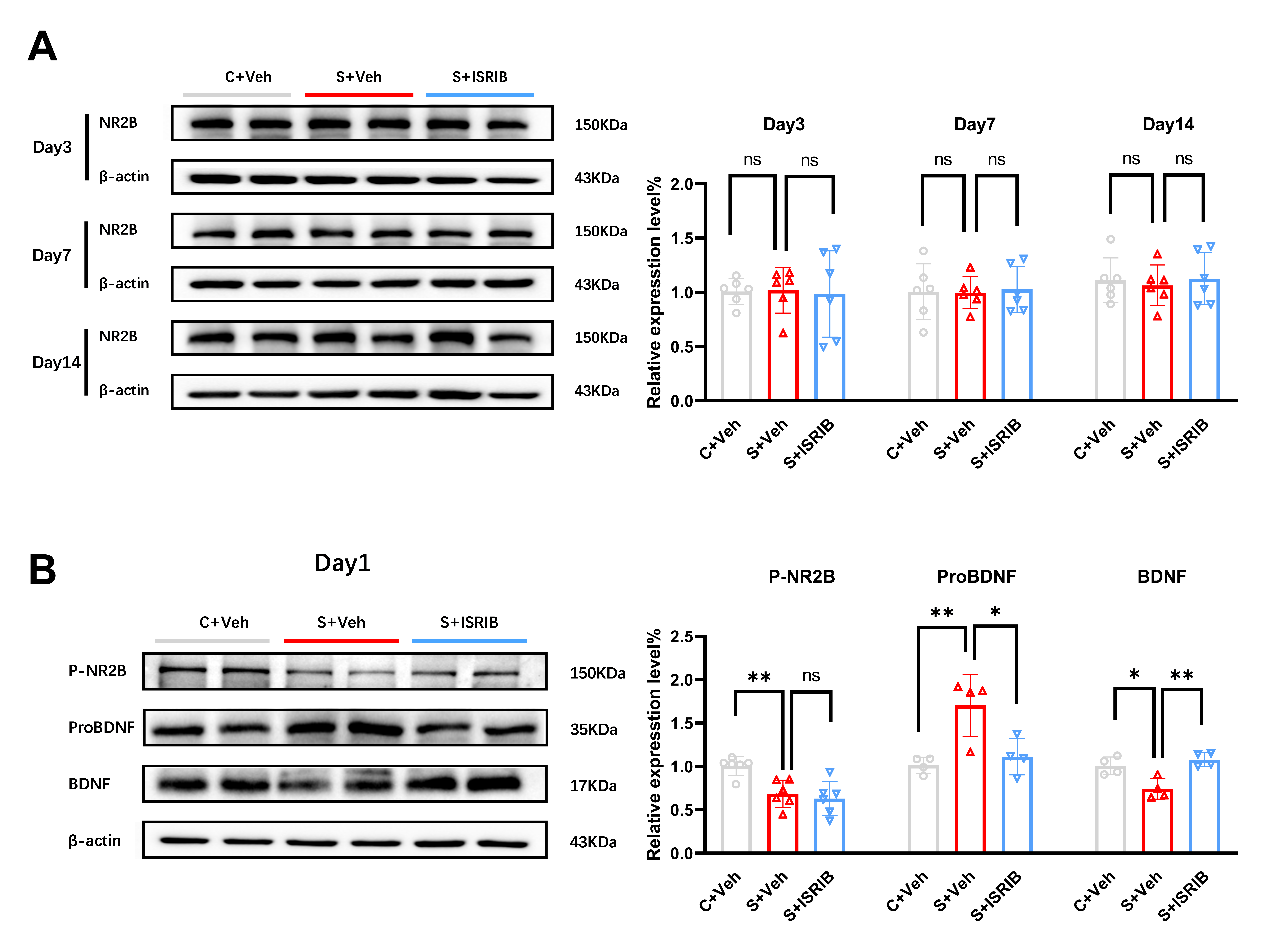


**Supplemental Figure 5.**

(A) Changes of NR2B protein expression at post-surgery day 3,7 and 14 in all groups. Representative protein bands shown on the left and quantification shown on the right (Day3:n=6,Day7:n=6,Day14:n=6). (B) Changes of P-NR2B, BDNF, and proBDNF protein expression at post-surgery day 1 in all groups. Representative protein bands shown on the left and quantification shown on the right (P-NR2B:n=6,ProBDNF:n=4,BDNF:n=4). Data are presented as the mean ±SEM. One-way ANOVA was used for statistical analysis. *p<0.05; **p<0.01; ***p<0.001.
